# Morphological predictors for microsatellite instability in urothelial carcinoma

**DOI:** 10.1186/s13000-021-01168-2

**Published:** 2021-11-20

**Authors:** Eduardo Sobrino-Reig, Telma Meizoso, Jesús García, David Varillas-Delgado, Yasmina B. Martin

**Affiliations:** 1 Hospital Universitario del Tajo, Aranjuez, Madrid, Spain; 2 Hospital Universitario de Móstoles, Móstoles, Madrid, Spain; 3 Facultad de Medicina, Universidad Francisco de Vitoria, 28223, Pozuelo de Alarcón, Madrid, Spain; 4 Facultad de Ciencias de la Salud, Universidad Francisco de Vitoria, Pozuelo de Alarcón, Madrid, Spain

**Keywords:** Cancer, Mismatch repair, Immunohistochemistry, Screening protocol, Tissue microarray

## Abstract

**Introduction:**

Microsatellite instability occurs due to a series of mutations in the DNA pairing error repair (Mismatch repair; MMR) genes, which can affect germ cells as occurs in Lynch syndrome, whose patients are at high risk of developing multiple cancers. The loss of MMR protein is commonly determined by immunohistochemical studies. Although the relation between microsatellite instability and urothelial carcinomas has been widely studied, its evaluation is not currently performed in the analysis of urothelial carcinomas.

**Methods:**

In this study, the microsatellite status of 139 urothelial carcinomas was analyzed and their clinicopathological characteristics were evaluated. We identified that 10.3% (13 patients) of urothelial carcinomas had loss of MMR protein expression (9 MLH1; 5 MSH2; 2 PMS2; 2 PSH6; *n* = 139).

**Results:**

Results suggest that these tumors occur more frequently in males, are more frequently located in the bladder or ureters, and present a high tumor grade with a papillary histological pattern that does not infiltrate the lamina propria or, in the case of infiltrating tumors, that grows into perivesical tissues.

**Conclusions:**

We identified patients with the aforementioned tumor characteristics as patients with a high probability of presenting loss of MMR protein expression, and consider that only these patients should undergo further immunohistochemical and molecular techniques for proper diagnosis. Therefore, we propose that the clinicopathological characteristics found in the present study could become possible markers to determine which cases should undergo additional tests.

## Introduction

Cancer stands as one of the leading public health issues of our times. Currently, one in three women and one in two men will develop a tumor during their lifetime. The increase in cancer prevalence has been related to the aging and growth of the population and improvements in life expectancy [1].

Urothelial carcinoma is the twelfth most common cancer worldwide [2], and the fifth most common cancer in Spain [3]. Estimates indicate a global incidence of 573,278 new cases per year, which cause approximately 212,536 annual deaths [2]. The incidence of urothelial carcinoma in Spain is estimated in 21,093 people per year [3].

Previous molecular genetic studies have shown a relationship between urothelial carcinomas and microsatellite instability (MSI). Namely, between 1.1 and 28% of urothelial carcinomas present MSI [4–14]. MSI is caused by mutations in the genes of the DNA repair system, known as Mismatch repair (MMR) [11], which fail to repair DNA duplication errors. This leads to an accelerated and indiscriminate accumulation of mutations in nucleotides through insertions or deletions [5, 15]. Moreover, germline mutations in DNA mismatch repair genes predispose to several gastrointestinal, urologic, gynecologic, and skin tumors at younger ages, also known as Lynch syndrome [6]. Carcinogenesis in patients with Lynch syndrome is due to an accumulation of somatic reading frame mutations within microsatellite regions in genes that control growth and apoptosis [4, 5, 11, 15].

The first test to examine the loss of MMR proteins is immunohistochemistry (IHC), which is currently only recommended for colorectal [16–19] and endometrial carcinomas [20–22]. This technique is not performed in urothelial carcinomas, although its relationship with MSI has been widely studied [9]. In fact, urothelial carcinomas with MSI highly benefit from adjuvant cisplatin-based chemotherapy [23], as well as the use of antibodies against CTLA4 and PD-1 [24]. Patients with urothelial carcinomas with identified MSI could also benefit from prophylactic treatment based on the use of Acetylsalicidal acid [25, 26], hormone replacement therapy [27], ibuprofen or calcium supplements [28, 29]. The importance of identifying patients with urothelial carcinomas with MSI is becoming more and more evident, as the clinical differences require specific treatments for better prognosis. Therefore, pathologists have an increasingly relevant role in the care of these patients [30].

Several authors, such as Ju [9], Joost [5] and Harper [8] and collaborators, have shown a correlation between the clinical and histological characteristics of urothelial carcinomas and the presence of MSI. Urothelial carcinomas with MSI are usually high-grade papillary tumors without the presence of marked nuclear pleomorphism, in stages pTa or pT1, with the presence of intratumoral lymphocytosis (20 lymphocytes per 10 HPF). They occur more frequently in men between 36 and 90 years of age and are mainly located in the bladder, although they can be found in any location lined by urothelial mucosa.

The aim of this study is to identify patients with urothelial carcinomas with presence loss of MMR protein expression. Although it is already known that carrying out an IHC and molecular study would allow the diagnosis of all patients, the economic cost is too high. Therefore, we propose to use the histological characteristics of the tumor and the clinical data of the patient as a guide to classify patients as in “high” or “low risk of presenting loss of MMR protein expression” as a first approach to narrow down the number of patients that are submitted to take additional tests. In this sense, only patients classified as “high risk of presenting loss of MMR protein expression” would continue with *polymerase chain reaction* (PCR) analysis. This system would allow all patients to receive a correct screening with a single histological section stained with Hematoxylin - eosin (H&E).

## Materials and methods

### Study design and case selection

An observational study was carried out by reviewing cases of urothelial carcinoma. Clinical data, histological sections and IHC stains of urothelial tumors from the University Hospital of Móstoles, (Madrid, Spain) were used.

**Fig. 1:**
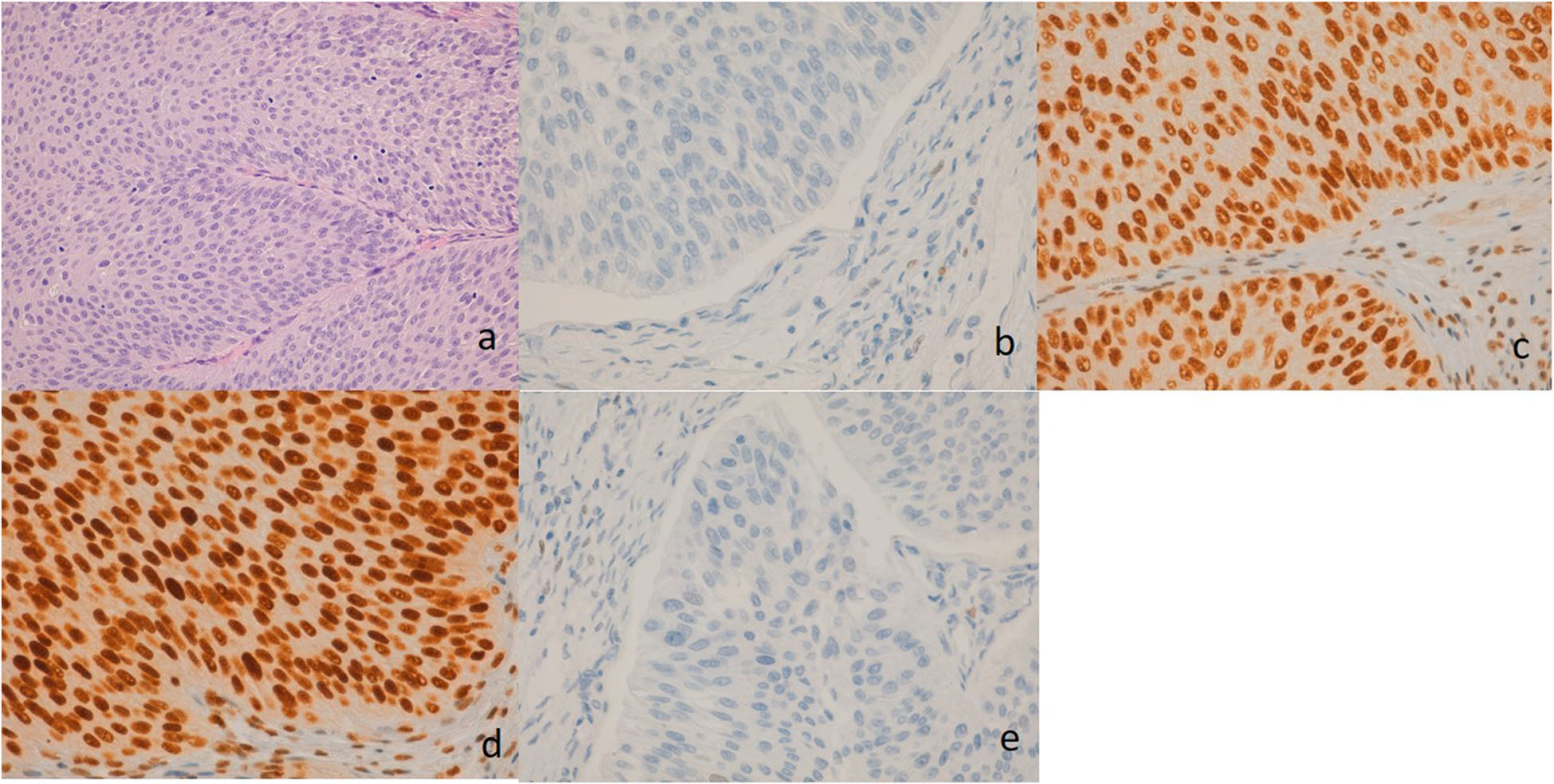
Photomicrographs to illustrate the inmunohistochemistry for mismatch repair protein. Hematoxylin & eosin stain of the tumor (a). Absent nuclear staning of MLH1 (b). Intact nuclear expression of PMS2 (c). Absent nuclear staning of MSH2 (d). Intact nuclear expression of MSH6 (e). Magnification: (a) 20x; (b-e) 40x

During January 2013 to 2014, 139 consecutive cases with urothelial carcinoma, which had been diagnosed by the Pathological Anatomy Service of the University Hospital of Móstoles, were selected (Fig. 1). Based on previously performed studies [5, 6, 11], a Type II error of 20% (80% power) with the sample size of 139 patients was supposed to provide enough statistical power according to previous studies [5, 6, 9, 11].

All the data used and obtained were coded and treated anonymously throughout the study. The study was approved by the Research Committee of the University Hospital of Móstoles, (Madrid, Spain) (No. org / int 005/2018).

### Inclusion and exclusion criteria

To avoid bias in the selection of cases, samples from patients with a tumor coded as “urothelial carcinoma” were included in the study, accepting any location or stage with available material. Urothelial carcinoma samples smaller than 0.3 cm in size were excluded from the study to avoid consuming the entire sample. They may be relevant for future studies. In the same way, tumors with large geographic areas of necrosis (necrotic tumor) were not included.

### Clinical analysis

Patients were classified according to their gender (male or female), age (in 5 groups: Group 1: 0–49 years; Group 2: 50–59 years; Group 3: 60–69 years; Group 4: 70–79 years Group 5: 80–99 years) and tumor location at the time of diagnosis (bladder, urethra and ureters).

### Pathologic analysis

Tumors were analyzed in blind conditions by a single pathologist. A representative block of urothelial carcinoma was received from the Anatomic Pathology Service of the University Hospital of Móstoles, and sections stained with hematoxylin and eosin-stained (H&E) were prepared. The H&E sections were reviewed, and the following histological criteria were used to evaluate the tumors:

#### Histology of the tumor

The tumors were classified according to the histological pattern they presented, classifying the urothelial carcinoma according to the following subtypes:

- Urothelial carcinoma with solid nests: presents an architecture in small and irregular nests or forming abortive tubules. Sometimes the nests anastomose, giving images of cords or trabeculae [4].
- Papillary urothelial carcinoma: presents fine fibrovascular axes lined by neoplastic urothelial tissue of variable thickness, they can present extensive ramifications. Neoplastic cells present abnormalities in size, shape, and nuclear chromatin and abnormalities in cell orientation [4].
- Mixed urothelial carcinoma: Urothelial carcinoma in which more than one pure pattern is found. Urothelial carcinoma with squamous differentiation will not be assessed within this classification, considering them as an own entity [4, 31].
- Urothelial carcinoma with squamous differentiation: Urothelial carcinoma with the presence of nests of malignant squamous epithelium, characterized by polygonal cells and evidence of keratinization or intercellular bridging. The presence of dyskeratosis and corneal pearls can be identified [4].

#### Classification of tumor staging

The classification was conducted according to the “Protocol for the Examination of Specimens From Patients With Carcinoma of the Urinary Bladder” the 8th Edition, AJCC Staging Manual, June 2017 [32] (Table 1).

**Table 1:** Tumor classification according to the grade of invasion as defined in [32]

Stage	Definition
Ta	Papillary noninvasive carcinoma.
Tis	Flat urothelial carcinoma in situ.
T1	Tumor invades lamina propia. *Connective tissue between the urothelium and the detrusor muscle.*
T2	Tumor invades muscularis propria. *Thick bundles of detrusor muscle.*
T3	Tumor invades perivesical tissue. *Adipose tissue beyond muscularis propia.* *T3a: microscopic invasion.* *T3b: macroscopic invasion.*
T4	Tumor invades other organs.

#### Histologic grade

The tumors received a single grade of differentiation (high or low) according to the criteria included in the “The World Health Organization/International Society of Urological Pathology” [33] This criteria has been endorsed by the “AJCC Staging Manual” [32] for standardized cancer.

#### Tumor necrosis or evident apoptosis

Tumors were evaluated for the presence or absence of necrosis. The tumor was classified as negative if the focus of necrosis was less than 10%.

Samples were classified into two groups: with or without tumor necrosis or apoptosis.

#### Presence of nucleolus

The presence or absence of nucleolus in the nuclei was examined. When a nucleolus was identified, it was classified according to its intensity. Samples were classified in three groups defined as follows: G0: nucleolus is not identified or not evident; G+: evident nucleolus is identified at 400x magnification; G++: obvious nucleolus is identified at 200x magnification.

#### Growth pattern

The growth pattern of the tumor was examined, and two groups were established: “Growth pattern with exophytic characteristics”, for tumors which show an outward growth, that is, towards the bladder or towards the tubular lumens in the case of tumors located at the level of the ureter or urethra; and “growth pattern of endophytic characteristics”, which included tumors that grow towards the chorion.

#### Lymphovascular invasion

The presence of tumor urothelial cellularity inside lymphatic and blood vessels was examined. They were classified into two groups: a first group for tumors in which there is no evidence of invasion in the lymphatic vessels or blood vessels, and a second group in which lymphovascular invasion can be identified.

#### Perineural invasion

When studying the nerves, the presence of urothelial carcinoma was observed in different locations, such as within the perineurium, within the nerve, surrounding the nerve or invading ganglionic tissue. Tumors were classified in two groups, according to whether perineural invasion was present or absent.

#### Presence of peritumoral lymphocytes

The percentage of lymphocyte cellularity in five high-power fields was analyzed in the peritumoral stroma, preferably considering the areas with more lymphocytes (hot-spot). Tumors were classified in three blocks: Block P0: there is no lymphocytic infiltrate, less than 5%. Block P1: evidence of minimal lymphocytic infiltrate, between 5 and 15%. Block P2: presence of evident lymphocytic infiltrate, greater than 15%.

#### Presence of intratumoral lymphocytes

At the tumor level, the percentage of lymphocyte cellularity in five high-magnification fields of each sample was studied, preferably considering the areas that had more lymphocytes (hot-spot). Apoptotic cells were not counted. Classification was designed in three blocks: Block L0: there is no lymphocytic infiltrate, less than 5%; Block L1: evidence of minimal lymphocytic infiltrate, between 5 and 15%; Block L2: presence of evident lymphocytic infiltrate, greater than 15%.

### Microsatellite analyses

Two tissue microarrays were performed on the studied samples, including the most representative parts of the tumor. The construction of the tissue microarrays was carried out in the Histopathology Unit of the Spanish National Cancer Research Center (CNIO), Madrid. A total of 139 tumor tissue samples were included, each tumor is represented with two 1 mm tumor cores, in turn, 2 control tissue samples (tonsil tissue) were included in each tissue microarray. Sections between 2 and 5 μm of the tissue microarray blocks were made. Sections were deparaffinized and stained with H&E to verify the correct construction of the blocks and the representation of all cases, we consider representative, if it includes more than 200 tumor cells. Once the blocks were verified, sections between 2 and 5 μm were made to perform the immunohistochemical (IHC) techniques. The IHC techniques, were performed using antibodies against MutL Proytein Homolog 1 (MLH1), MutL Proytein Homolog 2 (MSH2), MutL Proytein Homolog 6 (MSH6), and Postmeiotic Segragation Increased 2 (PMS2). IHC hybridizations analyses were conducted at the Department of Anatomic Pathology of the University Hospital of Móstoles and at CNIO (Fig. 1).

After determining the status of the MMR proteins using one of the IHC techniques, the absence of expression of one or more MMR proteins will be classified as dMMR. To classify patients as high-grade microsatellite instability (MSI-H), it must be performed using a PCR-based analysis in which two to five microsatellite tumor foci are identified [19, 34].

Taking as a reference the studies carried out in colorectal carcinoma, depending on the lack of expression of each of the MMRs, the IHC study can predict the probability that the patient has Lynch syndrome or is a sporadic tumor and recommends a series of molecular studies, which will be of great interest for future research:

The loss of isolated nuclear expression of MLH1/PMS2 or the loss of MLH1 and PMS2, may be due to the methylation of the MLH1 promoter and / or the BRAF mutation (for tumors with this lack of IHC staining, direct tests of hypermethylation of the MLH1 promoter and / or BRAF V600E mutational analysis; both the absence of MLH1 methylation and the BRAF V600E mutation suggest the possibility of Lynch syndrome and therefore sequencing and / or germline deletion / duplication tests may be indicated) [19, 35].

Loss of MSH2 and MSH6 nuclear expression or isolated loss of MSH6 or PMS2 nuclear expression predicts a high probability of Lynch syndrome (large germline deletion / duplication and / or sequencing tests may be indicated) [19, 36].

## Statistical analysis

Statistical analysis was performed using the SPSS 21.0® package for Windows ((IBM Corp. Released 2012. IBM SPSS Statistics for Windows, Version 21.0. Armonk, NY: IBM Corp). Categorical variables were expressed by frequencies and percentages. Statistical inference was made using the Chi-square test for analysis of nominal variables, Mann-Whitney U test for comparison of ordinal variables and binary logistic regression for predictor variables, carrying out multivariate back-step analysis to define models. Results were considered statistically significant at a *P*-value < 0.05.

## Results

### Tumor and patient characteristics from the population sample of Móstoles

The demographic and clinicopathologic data of the 139 patients diagnosed with urothelial carcinoma are shown in Table 2. The average age at diagnosis was between 60 and 70 years. Diagnosis was slightly more frequent in women than men. The most common location of the tumor was the bladder (93.5%), followed by the urethra and ureter (4.3 and 2.3%, respectively).

**Table 2:** Patient and tumor characteristics of 139 patients diagnosed with “urothelial carcinoma”

Characteristic		% (N)
*Age*	*< 49 years*	5.7 (8)
	*50–59 years*	12.9 (18)
	*60–69 years*	39.6 (55)
	*70–79 years*	23. (33)
	*> 80 years*	18.0 (25)
*Sex*	*Male*	67.6 (94)
	*Female*	32.4 (45)
*Localization*	*Bladder*	93.5 (103)
	*Urethra*	4.3 (6)
	*Ureter*	2.3 (3)
*Histology of the tumor*	*Papillary*	56.8 (79)
	*Mixed*	28. (39)
	*Solid*	12.9 (18)
	*Urothelial carcinoma with squamous differentiation*	2.2 (3)
*Classification of tumor staging*	*No infiltration*	35. (49)
	*Infiltration of lamina propia*	49.6 (69)
	*Infiltration of muscle*	10.8 (15)
	*Infiltration of perivesical tissue*	3.6 (5)
	*Infiltration of other organs*	0.7 (1)
*Histologic grade*	*Low*	59.6 (80)
	*High*	42.2 (59)
*Tumor necrosis or evident apoptosis*	*Yes*	53.3 (74)
	*No*	46.7 (65)
*Growth pattern*	*Endophytic*	14.4 (20)
	*Exophytic*	85.6 (119)
*Presence of nucleolus*	*No*	54.0 (75)
	*Yes +*	22.3 (31)
	*Yes ++*	23.7 (33)
*Lymphovascular invasion*	*Yes*	2.2 (3)
	*No*	97.8 (136)
*Neural invasion*	*Yes*	0 (0)
	*No*	100 (139)
*Presence of peritumoral lymphocytes*	*0–5%*	46.8 (65)
	*6–30%*	33.2 (47)
	*> 30%*	20.0 (27)
*Presence of intratumoral lymphocytes*	*0–5%*	85.6 (119)
	*6–20%*	10.1 (14)
	*> 20%*	4.3 (6)

The microscopic study identified that the patients studied from the population of Móstoles presented in most cases a papillary histological pattern (79 patients out of 139), with a predominant exophytic growth of high tumor grade with necrosis and the presence of nucleolus in 46.1% of the cases. These tumors had a low presence of intratumoral lymphocytes and peritumoral lymphocytes. The study of the degree of tumor infiltration showed that in more than half of the cases the urothelial tumors were non-muscle invasive. Vascular invasion was identified in only 1 of the cases and perineural invasion in none of the samples studied.

### MMR protein expression study

After analyzing the characteristics of the tumors and confirming that they present similar characteristics to the general population, we divided tumor samples into two groups. The first group included patients with loss of MMR protein expression (no identified immunoreactivity in any of the genes involved in the study) and the other group included patients who did not present alterations at this level. Tumor sample classification resulted in the identification of 13 patients (10.3%) who presented loss of MMR protein expression and 126 (89.7%) who did not present MSI (Table 3).

**Table 3:** Patients with loss of MMR protein expression (+ indicates presence of loss of MMR protein expression by IHC)

	MLH1	PMS2	MSH2	MSH6
Patient 1	+		+	
Patient 2	+			
Patient 3	+		+	
Patient 4	+			
Patient 5	+			
Patient 6		+	+	+
Patient 7	+			
Patient 8	+			
Patient 9		+	+	+
Patient 10	+		+	
Patient 11			+	
Patient 12	+	+		+
Patient 13			+	

Results from the association analysis between patient/tumor factors and the presence or absence of loss of MMR protein expression are shown in Table 4. When assessing the frequency of mutation between patient genders, it was observed that loss of MMR protein expression was statistically more frequent in men (22.2%) compared to women (3.2%), showing a risk of loss of MMR protein expression in men with an Odd Ratio (OR) of 6963 (95% CI: 2.014–24.069; *p* < 0.001).

**Table 4:** Correlation between the presence or absence of loss of MMR protein expression with parameters of influence in the different tumors

Factor		Loss of MMR protein expression		*P-value*
		Yes	No	
*Age*	*< 49 years*	0.0%	100.0%	N.S.
	*50–59 years*	11.1%	88.9%	
	*60–69 years*	10.9%	89.1%	
	*70–79 years*	6.1%	93.9%	
	*> 80 years*	12.0%	88.0%	
*Sex*	*Male*	22.2%	77.8%	< 0.001
	*Female*	3.2%	96.8%	
*Localization*	*Bladder*	7.7%	92.3%	0.001
	*Urethra*	0.0%	100.0%	
	*Ureter*	50%	50%	
*Histology of the tumor*	*Papillary*	11.5%	88.5%	N.S.
	*Mixted*	5.3%	94.7%	
	*Solid*	11.8%	88.2%	
	*Urothelial carcinoma with squamous differentiation*	0.0%	100.0%	
*Classification of tumor staging*	*No infiltration*	14.3%	85.7%	0.006 (*LP*)
	*Infiltration of lamina propia*	1.4%	98.6%	< 0.001 (*TP*)
	*Infiltration of muscle*	13.3%	86.7%	N.S.
	*Infiltration of perivesical tissue*	40.0%	60.0%	N.S.
	*Infiltration of other organs*	0.0%	100.0%	N.S.
*Histologic grade*	*Low*	10.2%	89.8%	N.S.
	*High*	8.8%	91.2%	
*Tumor necrosis or evident apoptosis*.	*Yes*	5.4%	94.6%	0.088
	*No*	13.8%	86.2%	
*Growth pattern*	*Endophytic*	8.4%	91.6%	N.S.
	*Exophytic*	15.0%	85.0%	
*Presence of nucleolus*	*No*	13.3%	86.7%	N.S.
	*Yes +*	6.5%	93.5%	
	*Yes ++*	3.0%	97.0%	
*Lymphovascular invasion*	*Yes*	33.3%	66.7%	N.S.
	*No*	8.8%	91.2%	
*Neural invasion*	*Yes*	9.4%	90.6%	N.S.
	*No*	9.4%	90.6%	
*Presence of peritumoral lymphocytes*	*0–5%*	55.30%	44.40%	N.S.
	*6–30%*	39.10%	35.70%	
	*> 30%*	12.80%	19.80%	
*Presence of intratumoral lymphocytes*	*0–5%*	92.30%	84.90%	N.S.
	*6–20%*	0.00%	11.10%	
	*> 20%*	7.70%	4.00%	

*N.S.* No statistical significance

The age categories did not show statistical differences regarding loss of MMR protein expression. No loss of MMR protein expression was observed in patients under 49 years of age, whereas the rest of the age categories showed instability percentages between 6.1 and 12.0% (*p* = 0.795).

The ureters were statistically significantly the most common location in which loss of MMR protein expression was present, with 50% of the cases showing loss of MMR protein expression in this location (95% CI: 2137-67,380; *p* = 0.001). The OR risk of presenting loss of MMR protein expression in the ureters was 12,000 times the risk of presenting loss of MMR protein expression in the bladder or urethra.

Regarding tumor infiltration in the lamina propria, no statistically significant differences (*p* = 0.141) were observed between loss of MMR protein expression and the presence (6.9%) or absence (14.9%) of infiltration.

The study of the degree of tumor infiltration based on the tumor staging guidelines from “AJCC Staging Manual” [32] showed statistical differences between the risk of presenting loss of MMR protein expression between patients with tumors that do not infiltrate the lamina propria (pTa) and those patients with tumors that infiltrate the lamina propria (pT1), with an OR of 9.857 (95% CI: 1.253–77.575; *p* = 0.006). An OR of 102.00 (95% CI: 7.103–1464.69; *p* < 0.001) of presenting loss of MMR protein expression was found in patients with tumors that cause infiltration to perivesical tissues (pT3) compared to patients with tumors whose infiltration is limited to the lamina propria (pT1).

The most frequent tumor grade in both groups (presence and absence of loss of MMR protein expression) was “high grade”. No statistical differences were observed between presenting a high (8.8%) or low grade (10.2%) within the loss of MMR protein expression group (*p* = 0.776). Similarly, no statistical differences were observed within the loss of MMR protein expression group between tumor growth with endophytic characteristics (8.4%) and growth with exophytic characteristics (15.0%) (*p* = 0.349).

The rest of the parameters evaluated did not present statistically significant differences between carcinomas with and without loss of MMR protein expression (Table 4).

## Discussion

The histopathological identification of different phenotypic characteristics is correlated with a molecular classification, which is how classical surgical pathology can be linked with newer molecular analysis techniques [37, 38]. In our study, we demonstrated that urothelial carcinomas with potential loss of MMR protein expression can be identified by evaluating histopathological and clinical data of the patients.

The analysis of the clinical and histological characteristics of our tumors and their comparison with the general population [4, 7, 39, 40], suggested that our series is representative of urothelial carcinomas. This is because the variables studied presented the usual trends of urothelial tumors found in the general population.

Our research showed that 10.3% of urothelial carcinomas presented loss of MMR proteins expression, which were identified using IHC. Results showed that the loss of MMR protein expression most frequently occurs due to mutations in MLH1, followed by MSH2, PMS2 and MSH6. These results are in accordance with previous studies [5, 8–10, 41].

In our study sample, patients that presented urothelial carcinoma with loss of MMR protein expression, identified by IHC, apparently sporadic, were more frequently men and tumors were located in most cases at the level of the ureters and bladder. The histological study showed that these neoplastic proliferations presented a papillary pattern of high tumor grade that in most cases did not infiltrate the lamina propria or, in the case of infiltrating tumors, produced an infiltration into perivesical tissues.

Previous studies by Joost [5], Harper [8] and Ju [9] validate and expand our findings. These authors observed that patients with potential MSI have several characteristics in common. They are usually high-grade papillary tumors without the presence of marked nuclear pleomorphism in stages pTa or pT1, although they can be found in any tumor stage [5, 8, 9]. They are tumors that occur mainly in the ureters and bladder, although they can be found in any location lined by urothelial mucosa [5, 9]. Regarding the age of diagnosis, tumors with MSI occur more frequently in patients between 36 and 90 years old. This is in line with the results from our study, in which more than 61.5% of our patients presented tumors in stages pTa or pT1. The next most frequent tumor stage was pT3, which could be due to a late diagnosis of our patients, which led to a more advanced tumor stage. In relation to the presence of intratumoral lymphocytosis, our results differed from the study by Ju et al. [9]. The authors observed 20 lymphocytes per 10 HPF, whereas in our study very few patients presented intratumoral lymphocytes. This difference may be due to the design of the study, since we assessed intratumoral lymphocytosis in a hot-spot field, whereas Ju and collaborators counted the number of lymphocytes in 10 HPF. This difference in the design limits the comparison of results from the different studies.

On the other hand, we found differences in our results compared to previous studies. Urakami et al. [10] observed that tumors with MSI were more frequent in women, presented an inverted papillary pattern of low cytological grade and were diagnosed more frequently at pTa or pT1 stages. Our results differ at the gender, tumor pattern and grade levels. These differences may be due to the diversity of the population sample used, as Urakami et al. conducted their research in Tokyo, Japan. Dissimilarities between the European and Asian populations have been previously reported in other tumors, such as the squamous cell carcinoma of the lung, whose incidence in Europe is more frequent in men, whereas in Asia it occurs more frequently in women [42].

**Fig. 2:**
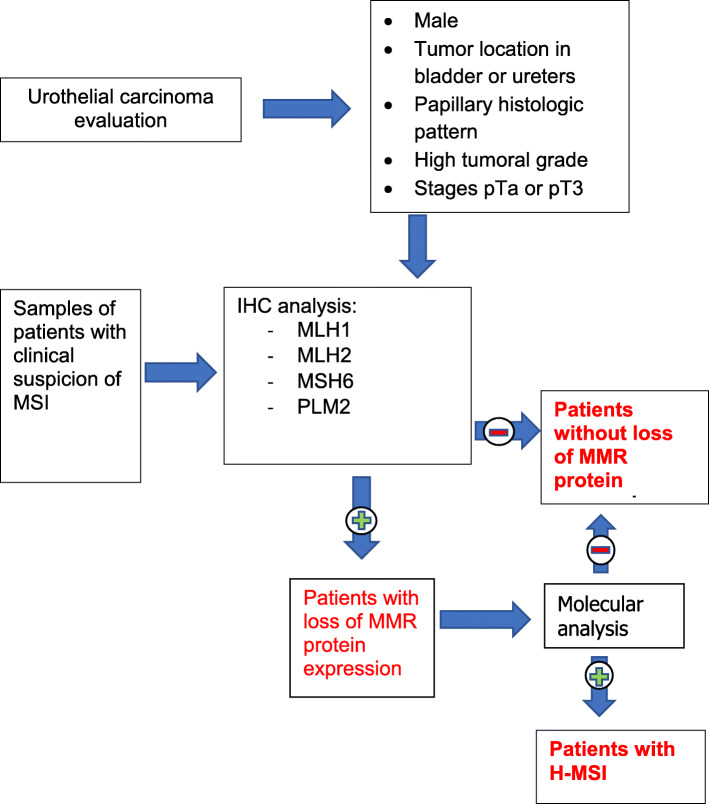
Screening protocol for the identification of patients with urothelial carcinomas with loss of MMR protein expression

Our study highlights that the identification of loss of MMR protein expression in urothelial carcinomas using a combination of histology data together with the clinical data of patients may provide an early detection tool for patient classification, and thus, a rapid tool for screening. It is also evidenced that evaluating histopathological and clinical data independently are not reliable enough to discriminate those patients with a higher probability of presenting loss of MMR protein expression. In this sense, we propose a protocol which integrates both data for patient classification (Fig. 2).

This study opens a way for future research, in which a comparison of the clinical and histological characteristics of patients who do not present IHC staining of one or more MMR proteins and the result of their PCR tests can be made together with the study for PD-L1. Due to the small size of most of the samples (mostly obtained by transurethral resection), it is considered important not to exhaust the sample of patients who participated in the study.

According to this protocol, once the patients with the highest probability of presenting loss of MMR protein expression have been identified, they should undergo immunohistochemical techniques for MMR protein expression evaluation. This screening reduces the number of requests for IHC techniques, since IHC will only be performed on those patients with clinical suspicion or those who meet the aforementioned histological and clinical characteristics. If patients do not express any of the IHC markers, a molecular study would be carried out. The advantages of using this screening system could include an early detection of patients with loss of MMR protein expression, a reduction of economic costs as fewer IHC tests would be conducted, and, in the long term, a reduction of the morbidity and mortality of patients.

## Conclusion

There is wide evidence that urothelial carcinomas present loss of MMR protein expression. In our study, the prevalence of urothelial carcinomas with loss of MMR protein expression was 10.3%. The combination of clinical data and histopathological characteristics may allow early identification of patients with high risk of presenting loss of MMR protein expression. Our study identified that these patients as male, with a tumor located in the bladder or ureters at the time of diagnosis, with a papillary histological pattern that does not infiltrate the lamina propria or, in the case of infiltrating tumors, that infiltrates perivesical tissues. We propose the evaluation of the clinicopathological characteristics identified in the present study to be applied as a screening guide, to help practitioners decide which cases should undergo additional tests. This protocol is prosed as a cost-effective tool that may help early diagnosis of patients with loss of MMR protein expression, reducing morbidity and mortality without implying an increase in work for the pathologist and laboratory technicians.

## Data Availability

Not applicable.

## Abbreviations

MMR: Mismatch repair
MSI: Microsatellite instability
IHC: Immunohistochemistry
HPF: High-power field
H&E: Hematoxylin – eosin
PCR: *polymerase chain reaction*
MLH1: MutL Proytein Homolog 1
MSH2: MutL Proytein Homolog 2
MSH6: MutL Proytein Homolog 6
PMS2: Postmeiotic Segragation Increased 2
